# Antibacterial Mechanisms and Antivirulence Activities of Oridonin against Pathogenic *Aeromonas hydrophila* AS 1.1801

**DOI:** 10.3390/microorganisms12020415

**Published:** 2024-02-19

**Authors:** Lunji Wang, Huijuan Li, Jinhao Chen, Yi Wang, Yuqing Gu, Min Jiu

**Affiliations:** 1 Key Laboratory of Microbial Resources Development and Utilization, Henan University of Science and Technology, Luoyang 471023, China; lunjiwang@haust.edu.cn (L.W.); 230320070648@stu.haust.edu.cn (J.C.); 2 College of Food and Bioengineering, Henan University of Science and Technology, Luoyang 471023, China; 220320070736@stu.haust.edu.cn (H.L.); 220320070732@stu.haust.edu.cn (Y.W.); guyuqing2001@163.com (Y.G.)

**Keywords:** *Aeromonas hydrophila*, oridonin, antibacterial efficacy, virulence factors, minimal inhibitory concentration

## Abstract

*Aeromonas hydrophila*, a Gram-negative bacterium widely found in freshwater environments, acts as a common conditional pathogen affecting humans, livestock, and aquatic animals. In this study, the impact of oridonin, an *ent*-kaurane diterpenoid compound derived from *Rabdosia rubescens*, on the virulence factors of *A. hydrophila* AS 1.1801 and its antibacterial mechanism was elucidated. The minimum inhibitory concentration (MIC) of oridonin against *A. hydrophila* AS 1.1801 was 100 μg/mL. Oridonin at inhibitory concentrations could significantly increase the electrical conductivity in the supernatant and escalate nucleic acid leakage (*p* < 0.01). This effect was concomitant with observed distortions in bacterial cells, the formation of cytoplasmic cavities, cellular damage, and pronounced inhibition of protein and nucleic acid synthesis. Additionally, oridonin at inhibitory levels exhibited a noteworthy suppressive impact on *A. hydrophila* AS 1.1801 across biofilm formation, motility, hemolytic activity, lipase activity, and protease activity (*p* < 0.05), demonstrating a dose-dependent enhancement. qRT-PCR analysis showed that the gene expression of *luxR*, *qseB* and *omp* were significantly downregulated after oridonin treatment in *A. hydrophila* AS 1.1801 (*p* < 0.05). Our results indicated that oridonin possessed significant antibacterial and anti-virulence effects on *A. hydrophila* AS 1.1801.

## 1. Introduction

*Aeromonas hydrophila* is an autochthonous inhabitant of aquatic ecosystems and has been recognized as a typical pathogen of human–animal–fish comorbidity [1,2]. It acts as a conditional pathogen in numerous aquatic species, including rainbow trout (*Oncorhynchus mykiss*), freshwater crocodiles, zebrafish (*Danio rerio*), Yesso scallop (*Patinopecten yessoensis*), and common carp (*Cyprinus carpio* L.) [1,2,3,4,5]. Under the pressure of increasing water temperature and deteriorating water quality, *A. hydrophila* will multiply in large numbers, often causing hemorrhagic septicemia in many marine and freshwater fish. Pathogenic *A. hydrophila* isolates are frequently detected in food items such as raw poultry, red meat, dairy, seafood, finfish, vegetables, and other items and can infect humans, resulting in watery diarrhea, septicemia and trauma infection [6].

Currently, antibiotics are commonly used to treat fish diseases caused by *A. hydrophila*. However, the unregulated and irrational use of antibiotics has led to an increasing problem of drug resistance in *A. hydrophila* and its cross-resistance to multiple drugs [7,8,9]. Therefore, the search for safe and effective antimicrobial agents has always been an important issue in aquaculture. Natural plant-derived antimicrobial substances have gained significant attention due to their safety, high efficacy, strong antioxidant properties, and broad spectrum of antimicrobial activity [10,11]. Chinese herbal medicine and its extracts have been extensively studied in the prevention and control of bacterial diseases in aquaculture. Numerous studies have demonstrated the potent antibacterial effects of various natural plant active ingredients against *A. hydrophila*, including cinnamaldehyde [12], resveratrol [13], carvacrol [14,15], and essential oils [16,17].

Recently, antivirulence therapy has attracted much attention and has been considered as an alternative to kill pathogens [18]. Antivirulence therapy involves interfering with the virulence factors or virulence-associated processes of pathogens to reduce their virulence capacity. *A. hydrophila* produces different virulence factors, including lipopolysaccharide (LPS), outer membrane proteins (OMPs), fimbriae, flagella, and other structural components, as well as extracellular mediators, including protease, lipase, hemolysins, biofilms, and quorum sensing (QS) communication, with all of these having the potential to contribute to host pathogenicity [19]. Pathogens can coordinate the formation of biofilm and the toxicity between virulence factors through QS [20].

Oridonin, an *ent*-kaurane diterpenoid compound derived from *Rabdosia rubescens*, has been found to possess various pharmacological activities, including anti-cancer, antibacterial, anti-inflammatory, and anti-oxidation effects [21,22]. For instance, studies have reported that oridonin can inhibit the growth of *Staphylococcus aureus* and the formation of its biofilm [23]. Additionally, our preliminary research found that oridonin exhibited strong inhibitory effects on *Bacillus subtilis*, *S. epidermidis*, *Listeria monocytogenes*, *Vibrio parahaemolyticus*, and *A. hydrophila* (unpublished data). However, limited information is available regarding the specific mechanism by which oridonin inhibits *A. hydrophila*. Exploring the impact of oridonin on *A. hydrophila* is critical for expanding its utility in aquaculture and prevention. Here, we detected the antimicrobial activity of oridonin and its impact on the growth, virulence-associated gene expression (*omp*, *luxR*, *hly*, *luxS*, and *qseB*), and activity of virulence factors (biofilm, protease, lipase, hemolysin, and swarming assays) in *A. hydrophila*.

## 2. Materials and Methods

### 2.1. Bacterial Strains and Chemical Agents

*Aeromonas hydrophila* AS 1.1801 was bought from the China Center for Type Culture Collection (CCTCC) and stored at −80 °C in nutrient broth (NB, Qingdao Hope Bio-Technology Co., Ltd., Qingdao, China) with 20% glycerol. The frozen strains were reactivated via inoculation in nutrient agar (NA, Qingdao Hope Bio-Technology Co., Ltd.) and incubated at 28 °C for 24 h. The isolated colonies were inoculated into 50 mL of NB and incubated under stirring at 180 rpm on a constant temperature shaker (Boxun, Shanghai, China) at 28 °C for 20 h. These bacterial inocula cultures (~1 × 10^8^ CFU/mL, 0.5 McFarland turbidity) were used for the antibacterial activity assays. Oridonin (˃98% HPLC purity; CAS no.28957-04-2) was bought from Shanghai Yuanye Bio-Technology Co., Ltd. (Shanghai, China) and dissolved in methanol to obtain a stock solution.

### 2.2. MIC Determination

The MIC of oridonin was determined via the broth dilution method, as described by Paudel et al. (2019) [24]. Oridonin was sterilized via filtration using a 0.22 μm millipore membrane filter attached to a syringe (Millipore, Nanjing, China). Two-fold dilutions of sterile oridonin ranging from 800 to 6.25 μg/mL were aseptically prepared in tubes of sterile NB. The tubes of NB were each inoculated with 50 μL of overnight (20 h) cultures of *A. hydrophila* AS 1.1801 to obtain an initial viable cell concentration of 5.0 Log CFU/mL. The experiment included positive (inoculated tubes, free of oridonin) and negative (non-inoculated tubes, free of oridonin) controls. All tubes were incubated at 28 °C and observed for turbidity after 24 h. The MIC was the lowest oridonin concentration at which no visible growth (absence of turbidity) occurred. Tests for MIC were performed in triplicates.

### 2.3. Growth Assay

The effect of subinhibitory concentrations of oridonin on *A. hydrophila* AS 1.1801 was determined spectrophotometrically using microplate readers (Infinite F50, TECAN, Männedorf, Switzerland). Concisely, overnight cultures of *A. hydrophila* AS 1.1801 were inoculated into NB broth without or with oridonin at different concentrations (0, 3.125, 6.25, 12.5, 25, 50, 100, and 200 μg/mL) at 28 °C for 26 h (stationary phase) under the condition of shaking at 180 rpm. At each of the time points viz. 0, 2, 4, 6, 8, 10, 12, 24, and 26 h, 200 μL of the oridonin-treated or untreated inoculum was added to a 96-well plate and determined for optical density (*OD*) at 600 nm. The experiments were repeated three times.

### 2.4. Cell Membrane Permeability Assay

#### 2.4.1. Determination of Conductivity

The permeability of bacterial membranes is expressed in the relative electric conductivity and determined according to the method described by Diao et al. (2014) [25]. After incubating at 28 °C for 20 h, *A. hydrophila* AS 1.1801 was separated via centrifugation at 8000 rpm for 10 min. Then, the bacteria were washed with 5% glucose until their electric conductivities were near to that of 5% glucose, and they were the case for isotonic bacteria. Oridonin at five different concentrations (0, 1/4, 1/2, 1, and 2 × MIC) was added to 5% glucose, and the electric conductivities of the mixtures were marked as *L*_1_. Then, different concentrations of oridonin were added to the isotonic bacteria solution. After completely mixing, the samples were incubated at 28 °C for 6 h, and then the conductivities were measured and marked as *L*_2_. The conductivity of bacteria in 5% glucose treated in boiling water for 5 min was served as the control and marked as *L*_0_. The permeability of the bacteria membrane is calculated according to the formula, with the relative electric conductivity (%) = 100 × (*L*_2_ − *L*_1_)/*L*_0_.

#### 2.4.2. Determination of Nucleic Acid Release

Nucleic acids (DNA and RNA) released from the cells of *A. hydrophila* AS 1.1801 after treatment with oridonin at different concentrations (0, 1/4, 1/2, 1, and 2 × MIC) were analyzed according to the method described by Kang and Song (2019) [26], with the introduction of some modifications. Overnight cultures grown on an NB were used for making a bacterial suspension in physiological saline (0.85% NaCl) and further adjusted to 0.5 McFarland turbidity using a McFarland densitometer (DEN-1, BIOSAN, Riga, Latvia). Inoculum were collected via centrifugation (8000 rpm, 4 °C for 10 min), washed with physiological saline, mixed with 20 mL physiological saline containing different concentrations of oridonin, and cultured at 28 °C for 3 h, 6 h, and 9 h under shaking conditions at 180 rpm. After incubation, the cell pellets treated with or without oridonin were harvested via centrifugation, as described above. The supernatant was collected, and its absorbance at 260 nm was measured to determine the amounts of intracellular DNA and RNA released from the cells of *A. hydrophila* AS 1.1801 after oridonin treatment. The supernatant obtained from the untreated *A. hydrophila* AS 1.1801 inoculum was used as a control.

### 2.5. Determination of Intracellular Total Protein

According to the method described by Kang and Song (2019) [26] with some modifications, the cultures of *A. hydrophila* AS 1.1801 (28 °C, 180 rpm for 12 h) were centrifuged at 11,000 rpm for 10 min under 4 °C. The supernatant was discarded, and the precipitated cells were washed twice with sterile phosphate buffer (pH 7.2), which was further adjusted to 0.5 McFarland turbidity using the McFarland densitometer. In this study, 100 mL of cell suspension was incubated at 28 °C under agitation of 180 rpm for 3 h, 6 h, and 9 h in the presence of oridonin at five different concentrations (0, 1/4, 1/2, 1, and 2 × MIC). Then, 25 mL of the samples with the same *OD*_600_ value was collected and centrifuged at 11,000 rpm for 10 min. The obtained cell pellets were washed thrice with sterile phosphate buffer (pH 7.2) and then electrophoretic loading buffer (2 mL) was added. The samples were heated for 10 min in a boiling water bath and were centrifuged at 11,000 rpm for 10 min under 4 °C. The protein content was determined with the bicinchoninic acid (BCA) Protein Assay Kit (C503021, Sangon Biotech (Shanghai) Co., Ltd., Shanghai, China). The experiment was repeated three times with appropriate untreated control.

### 2.6. Determination of DNA and RNA Synthesis

The determination of DNA and RNA synthesis was carried out according to the method described by Yuan et al. (2019) [23] with some modifications. The cultures of *A. hydrophila* AS 1.1801 (28 °C, 180 rpm for 12 h) were inoculated into NB medium with 2% inoculum. The cell suspension was incubated at 28 °C under the agitation of 180 rpm for 26 h in the presence of oridonin at five different concentrations (0, 1/4, 1/2, and 1 × MIC) and was centrifuged at 8000 rpm for 10 min under 4 °C. The obtained cell pellets were resuspended with sterile normal saline, fixed on a microscope slide, and treated with 4’, 6-Diamidino-2-phenylindole dihydrochloride (DAPI). The cells were incubated at 25 °C for 20 min in the dark, and the DNA fluorescence was observed under a fluorescence microscope (OLYMPUS, Tokyo, Japan).

### 2.7. Analysis of Morphological and Physical Changes

*A. hydrophila* AS 1.1801 was cultivated in NB until reaching an *OD*_600_ value of 0.5 at 28 °C under 180 rpm agitation and then treated with oridonin (1 × MIC) for 6 h. The bacteria were then centrifuged (5 min at 7000 rpm, 4 °C). The precipitated cells were washed three times with 0.1 M PBS (pH 7.4) for 15 min each again and post-fixed with 2.5% (*v*/*v*) glutar–aldehyde in 0.1 M PBS overnight at 4 °C. After this, the cells were dehydrated using sequential exposure per ethanol concentrations of 30%, 50%, 70%, 80%, 90% and 100% for 10 min, respectively. Embedding medium was then added to all the samples. Stained bacteria were utilized for transmission electron microscopy (TEM) analysis (HT7700, Hitachi, Ltd., Tokyo, Japan). Control analyses were conducted using untreated bacteria.

### 2.8. Effect of Oridonin on A. hydrophila Virulence

#### 2.8.1. Lipase Assays

As per a slightly modified version of a protocol published by Yang et al. (2014) [27], overnight cultures (4 μL of 0.4 *OD*_600nm_) were inoculated into the center of NA plates supplemented with 1% Tween80 (Sangon Biotech, Shanghai, China) and varying concentrations (1/16 × MIC–2 × MIC) of oridonin. After incubation for 48 h at 28 °C, the appearance of opalescent regions surrounding these colonies was assessed, and the zone diameters were measured.

#### 2.8.2. Swarming Assays

The swarming motility assays were examined by following the method of Jahid et al. (2015) [20]. Swarming motility was assessed using NB with 0.5% (*w*/*v*) agar and the respective oridonin at a final concentration of 1/16 × MIC − 2 × MIC. These plates were inoculated with the prepared bacterial suspensions in spots and were incubated for 48 h at 28 °C, and the diameters of the swarming motility zones were measured.

#### 2.8.3. Protease Assays

An azocasein assay was used to analyze protease activity based on a slightly modified version of a protocol from Chu et al. (2014) [28]. Briefly, 150 μL of *A. hydrophila* AS 1.1801 culture supernatant from samples treated with oridonin or not was combined with 0.3% azocasein (Sigma, Shanghai, China) in 1 mL of 0.05 M Tris-HCl and 0.5 mM CaCl_2_ (pH 7.5), followed by a 30 min incubation at 37 °C. Trichloroacetic acid (TCA) (10%, 500 μL) was added to terminate this reaction, after which the samples were centrifuged at 12,000 × g for 10 min under 4 °C. Then, 100 μL of the clear supernatant was added to 100 μL of 1 mol/L NaOH solution to neutralize TCA in a 96-well plate, and the absorbance was assessed at 400 nm in microplate readers (Infinite F50, TECAN, Männedorf, Switzerland).

#### 2.8.4. Hemolysis Activity Assays

Hemolysis assays were carried out as described by Luo et al. (2016) [29] with slight modifications. The cultures of *A. hydrophila* AS 1.1801 (28 °C, 180 rpm for 24 h) were centrifuged at 11,000 rpm for 10 min under 4 °C. The supernatant was then filtered using a 0.22 μm filter to obtain the cell-free supernatant. Sheep blood (Solarbio Science & Technology Co., Ltd., Beijing, China) was prepared by washing three times with PBS. Then, 100 μL of cell-free supernatant was added to 100 μL of 4% erythrocyte suspension and 800 μL sterile saline and incubated at 37 °C for 30 min with gentle shaking, followed by centrifugation at 5000 rpm for 10 min under 4 °C. The released hemoglobin was measured by *OD*_540_. The percentage of total hemolysis was calculated by comparing the *OD*_540_ of the samples with positive (100% lysis by 1% Triton X-100) and negative controls.

#### 2.8.5. Antibiofilm Activity Assays

A crystal violet (CV) assay in a 96-well plate was used to assess biofilm biomass inhibition by following the method of Cui et al. (2020) [30]. Overnight cultures of *A. hydrophila* AS 1.1801 were diluted 100 × in NB broth followed by culture in the presence or absence of oridonin (4 × MIC, 2 × MIC, 1 × MIC, 1/2 × MIC, 1/4 × MIC, and 1/8 MIC) in a 96-well plate for 24 h at 28 °C without shaking. The supernatant was removed from the cells, and then the wells were washed thrice with PBS and fixed with methanol for 15 min. Following the air-drying of plates at room temperature for 5 min, 1% CV was used to stain the plates for 20 min, followed by washing twice with PBS. De-staining was then achieved by adding 200 μL 30% glacial acetic acid, and the absorbance was measured at *OD*_562nm_. Percentage biofilm biomass inhibition was defined as [Control *OD* − Test *OD*/Control *OD*] × 100.

Cell viability was quantified via a modified 3-[4,5-dimethylthiazol-2-yl]-2,5-diphenyltetrazolium bromide reduction (MTT) assay as per Mu et al. (2014) [31]. The percentage inhibition of live bacteria was defined as [Control *OD* − Test *OD*/Control *OD*] × 100.

For fluorescence microscopy, the biofilm was cultured on glass coverslips (14 mm × 14 mm) at 28 °C for 24 h in a 24-well plate supplemented with 1 mL of NB with or without oridonin (2 × MIC, 1 × MIC, 1/2 × MIC, and CK). Fluorescence staining and observation were carried out according to Mu et al. (2014) [31]. Scanning electron microscopy (SEM) was conducted as described by Cui et al. (2020) [30].

### 2.9. qRT-PCR

*A. hydrophila* AS 1.1801 was grown overnight and diluted to an *OD*_600_ of 0.3. The bacterial cultures (1%, *v*/*v*) were inoculated into NB medium with or without oridonin and incubated at 28 °C for 24 h. The cells were harvested, and the RNA was extracted with a bacterial total RNA rapid extraction kit (Sangon Biotech (Shanghai) Co., Ltd., Shanghai, China), as per the provided directions. A nanodrop spectrophotometer (ND-2000, Nanodrop Technologies) was utilized to measure the quantity and quality of prepared RNA, while gel electrophoresis was used to assess its integrity. Using the *rpoB* gene as the internal reference control [32,33], the relative expression levels of *hly*, *omp*, *luxR*, *luxS,* and *qseB* genes were detected. qRT-PCR was conducted with 2 × SYBR Green qPCR Master Mix (High ROX) (Wuhan Servicebio Technology Co., Ltd., Wuhan, China). The primer sequences are listed in Table 1. All of the samples were analyzed in triplicate. The relative expression levels were calculated using the 2^−ΔΔCT^ method, where ΔΔCT = ΔCT (treated sample) − ΔCT (untreated sample), ΔCT = CT (target gene) − CT (*rpoB*), and CT is the threshold cycle value for the amplified gene.

### 2.10. Statistical Analysis

One-way ANOVA with Duncan’s multiple range test for cell membrane permeability, extracellular nucleic acid content, intracellular soluble protein content, lipase, protease, swarming motility, biofilm, hemolytic activity and Student’s test for virulence gene expression were performed using SPSS software (version 18.0, IBM-SPSS Inc., Armonk, NY, USA). A value of *p* < 0.05 and *p* < 0.01 was considered statistically significant. All experiments were carried out in triplicate, and all data were presented as mean ± standard deviation (SD).

## 3. Results

### 3.1. Determination of MIC of Oridonin against A. hydrophila AS 1.1801

**Figure 1:**
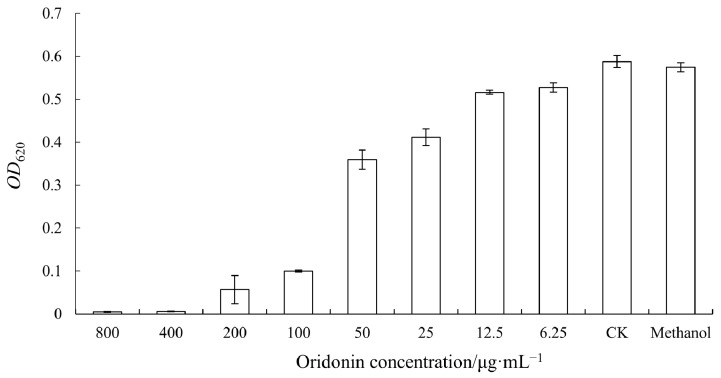
Determination of MIC of oridonin against *A. hydrophila* AS 1.1801. Notes: Control (CK): inoculated tubes, free of oridonin; Methanol: inoculated tubes containing the solvent methanol (<1%, *v*/*v*), free of oridonin.

The MIC of oridonin against *A. hydrophila* AS 1.1801 was found to be 100 μg/mL (Figure 1). Based on this fact, further experiments were performed to evaluate the impact of oridonin on the pathogenic factors of *A. hydrophila* AS 1.1801.

### 3.2. The Effect of Oridonin on the Growth of A. hydrophila AS 1.1801

**Figure 2:**
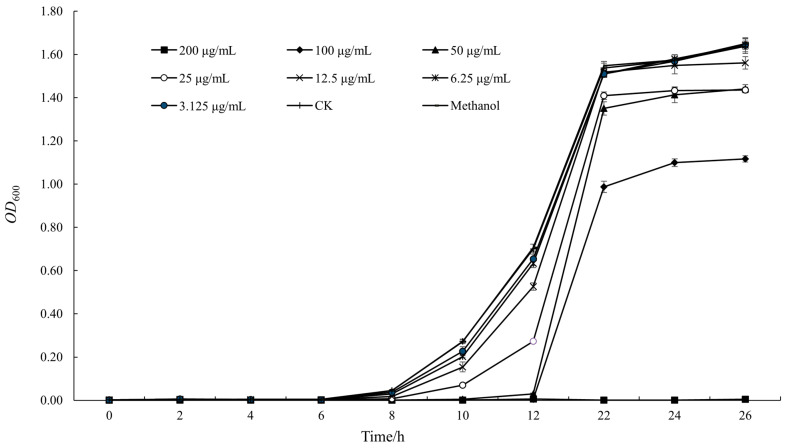
Effects of oridonin on the growth curve of *A. hydrophila* AS 1.1801.

The effect of oridonin on the growth of *A. hydrophila* AS 1.1801 is shown in Figure 2. As depicted in Figure 2, the subinhibitory effect of oridonin on *A. hydrophila* AS 1.1801 was an extension of the growth delay period and a reduction in the maximum growth rate. At a concentration of 2 × MIC, the growth of *A. hydrophila* AS 1.1801 was entirely inhibited by oridonin. Simultaneously, the results also showed that 1 × MIC oridonin did not completely inhibit the growth of *A. hydrophila* AS 1.1801 but did increase the lag time in the growth curve.

### 3.3. Antibacterial Mechanisms of Oridonin against A. hydrophila AS 1.1801

#### 3.3.1. Effect of Oridonin on the Cell Membrane Permeability

**Figure 3:**
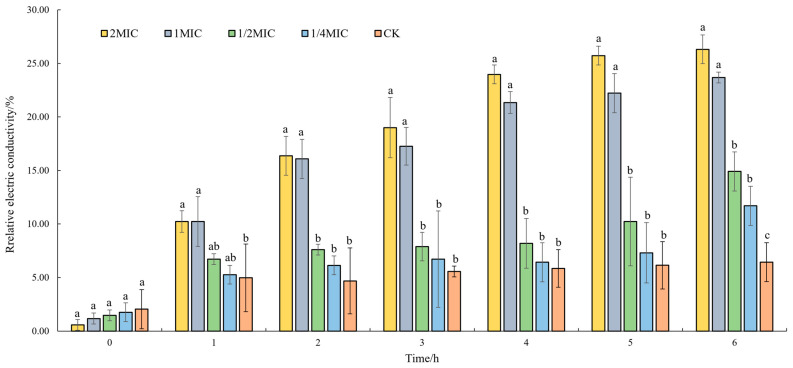
Effects of oridonin on the permeability of cell membrane of *A. hydrophila* AS 1.1801. Notes: Oridonin concentration: CK (0), 2 MIC (200 µg/mL), 1 MIC (100 µg/mL), 1/2 MIC (50 µg/mL), 1/4 MIC (25 µg/mL). Data are presented as the mean ± SD of three independent experiments. The results were analyzed with one-way ANOVA using Duncan’s multiple range test. a–c: Values with different letters are significantly different (*p* < 0.01), while those with similar letters are not.

To investigate the impact of oridonin on cell membrane permeability in *A. hydrophila* AS 1.1801, the relative electric conductivity was measured (Figure 3). The result illustrated that oridonin enhanced the conductivity of the cultures. The strains were treated with an inhibitory concentration of oridonin (1 × MIC and 2 × MIC) for 1 h. The relative electric conductivity of the oridonin-treated groups significantly increased compared to that at 0 h and was notably higher than the control group at 1 h (*p* ˂ 0.01). The relative electric conductivity of oridonin-treated groups was significantly higher, showing a significant deviation from the control group after 2 h (*p* ˂ 0.01). The relative electric conductivity of the cultures with sub-inhibitory concentration oridonin (1/4 × MIC and 1/2 × MIC) treatment significantly increased compared to that at 0 h. It was notably higher than the control group at 6 h for *A. hydrophila* AS 1.1801 (*p* ˂ 0.01). Additionally, the result revealed a slight increase in the relative conductivity of the control group after 1 h. This could be attributed to the dissolution of a small number of bacterial cells during the 6 h incubation period.

#### 3.3.2. Effect of Oridonin on the Integrity of Cell Membrane

Cell membrane integrity was assessed by measuring the release of cell constituents, including DNA and RNA, using absorbance at 260 nm in the bacterial supernatant. Changes in the amounts of intracellular DNA and RNA released from *A. hydrophila* AS 1.1801 cells after oridonin treatment at four time points (0 h, 3 h, 6 h, and 9 h) are presented in Table 2. The results illustrated that the amount of DNA and RNA released from *A. hydrophila* AS 1.1801 cells significantly increased after treatment with various oridonin concentrations for 3 h (*p* ˂ 0.01). However, the amount of DNA and RNA released from the cells did not notably increase with prolonged treatment time. Additionally, higher concentrations of oridonin resulted in increased DNA and RNA release from the cells, showing a significant difference between different treatment groups (*p* ˂ 0.01). These results implied that irreversible damage to the cytoplasmic membranes might occur, leading to the loss of cell constituents, including nucleic acid molecules and essential compounds, ultimately resulting in cell death.

### 3.4. Effect of Oridonin on the Intracellular Total Protein Synthesis

*A. hydrophila* AS 1.1801 bacteria were treated with oridonin at 1/4 × MIC, 1/2 × MIC, 1 × MIC, and 2 × MIC for 3, 6, and 9 h, respectively. Subsequently, changes in the intracellular proteins of the treated *A. hydrophila* AS 1.1801 bacteria were determined. The content of intracellular proteins in the oridonin-treated *A. hydrophila* AS 1.1801 was lower than that in the untreated control, indicating that the inhibitory concentration of oridonin (1 × MIC and 2 × MIC) may intervene in protein metabolism in the oridonin-treated groups (Table 3). In the subinhibitory concentration, the content of intracellular proteins in oridonin-treated *A. hydrophila* AS 1.1801 exhibited more variation and showed significant differences (*p* ˂ 0.05) than that in the untreated control.

### 3.5. Effect of Oridonin on the Synthesis of DNA and RNA

**Figure 4:**
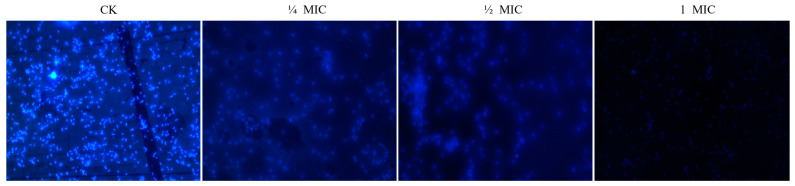
Effect of oridonin on intracellular nucleic acids synthesis of *A. hydrophila* AS 1.1801.

DAPI is a fluorescent dye that binds to DNA and RNA. Greater amounts of nucleic acid result in stronger fluorescence. DAPI can penetrate the cell membrane into the cell interior and bind with nucleic acid. The fluorescence microscope observation results of the effect of oridonin on *A. hydrophila* AS 1.1801 nucleic acid synthesis for 26 h are shown in Figure 4. The results demonstrated that the fluorescence intensity of *A. hydrophila* AS 1.1801 treated with the inhibitory concentration of oridonin was significantly weaker than that of the untreated oridonin groups. The effects of subinhibitory doses of oridonin on nucleic acid synthesis were also investigated. In this study, subinhibitory concentrations of oridonin exhibited a concentration-dependent inhibition of DNA synthesis. Compared to the control group, the fluorescence intensity of the oridonin treatment group gradually decreased with the increase in drug content.

### 3.6. Effect of Oridonin on the Morphological and Physical Changes of A. hydrophila AS 1.1801

**Figure 5:**
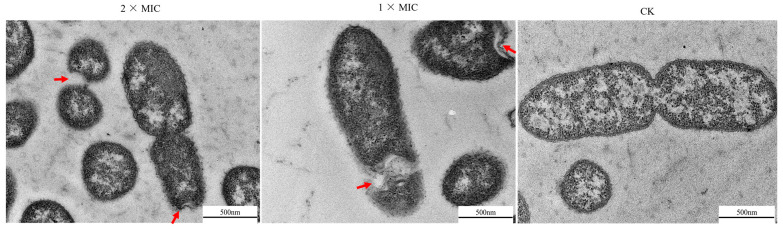
Morphology of *A. hydrophila* AS 1.1801 examined using transmission electron microscope (15,000×). The red arrows demonstrated the abnormal changes in *A. hydrophila* AS 1.1801.

*A. hydrophila* AS 1.1801 were treated with oridonin at 1 × MIC and 2 × MIC for 6 h. Subsequently, the morphological and physical changes in the treated bacteria were observed using TEM. As shown in Figure 5, the untreated cells exhibited short rod-shaped bacterium with clear boundaries between them. The cell structure was complete and clear in the control group, with evident and evenly distributed cytoplasm. Cells from the treatment group, on the other hand, exhibited severe deformation and displayed loss of intracellular material, cytoplasmic condensation, characteristic vacuolation, blurry constriction, and abnormal binary fission.

### 3.7. Inhibitory Effect of Oridonin on the Virulence of A. hydrophila AS 1.1801

**Figure 6:**
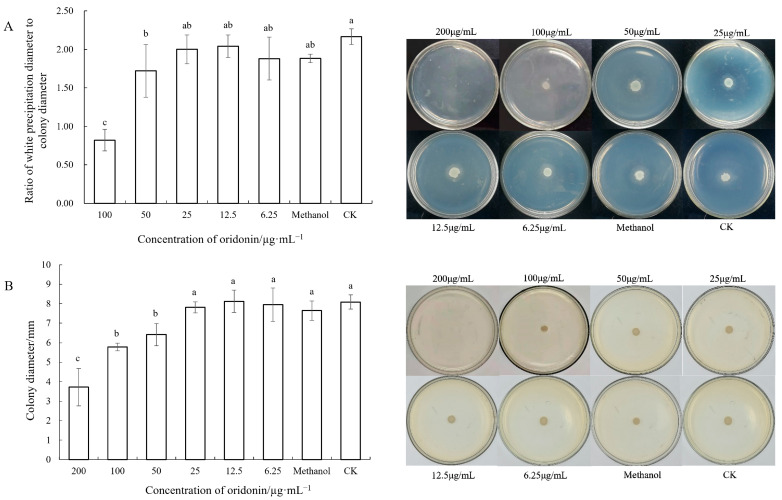
Effect of oridonin on lipase activity and swarming motility of *A. hydrophila* AS 1.1801. Notes: (**A**) lipase activity (left: data graph; right: experimental images); (**B**) swarming motility (**left**: data graph; **right**: experimental images). Data are presented as the mean ± SD of three independent experiments. The results were analyzed with one-way ANOVA using Duncan’s multiple range test. a–c: Values with different letters are significantly different (*p* < 0.05), while those with similar letters are not.

**Figure 7:**
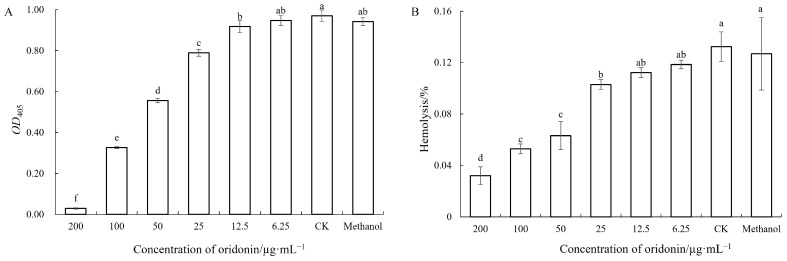
Effect of oridonin on protease and hemolytic activity of *A. hydrophila* AS 1.1801. Notes: (**A**) protease activity; (**B**) hemolytic activity. Data are presented as the mean ± SD of three independent experiments. The results were analyzed with one-way ANOVA using Duncan’s multiple range test. a–f: Values with different letters are significantly different (*p* < 0.05), while those with similar letters are not.

The initial experiments investigated the effects of oridonin on the levels of lipase and protease activity, hemolysin, and surface-associated swarming motility in *A. hydrophila* AS 1.1801. As shown in Figure 6 and Figure 7, the blank control group and the solvent control group showed no significant differences in lipase and protease activities, motility, and hemolysis of *A. hydrophila* AS 1.1801 (*p* > 0.05). This indicated that the solvent methanol did not affect the pathogenic factors of *A. hydrophila* AS 1.1801.

In Figure 6, compared to the control, the subinhibitory concentration of oridonin (<1/2 × MIC) showed no significant influence on lipase activity and motility of *A. hydrophila* AS 1.1801 (*p* > 0.05). However, when the concentration of oridonin exceeded 50 μg/mL, lipase and swarming activities significantly decreased. Compared to the untreated groups, the lipase and swarming activities of *A. hydrophila* AS 1.1801 treated with 1/2 × MIC were decreased by 20.7% and 20.8%, respectively. At higher concentrations (1 × MIC and 2 × MIC) of oridonin, a significant decrease was observed in the level of lipase and swarming activities compared with the subinhibitory concentration treatment groups (Figure 6A,B).

The impacts of oridonin on two other virulence factors (extracellular protease and hemolysin) were determined as well. The tests revealed that extracellular protease and hemolytic activity decreased with increasing oridonin concentration in a dose-dependent manner, with significant differences among different treatment groups (*p* ˂ 0.05) (Figure 7A,B).

**Figure 8:**
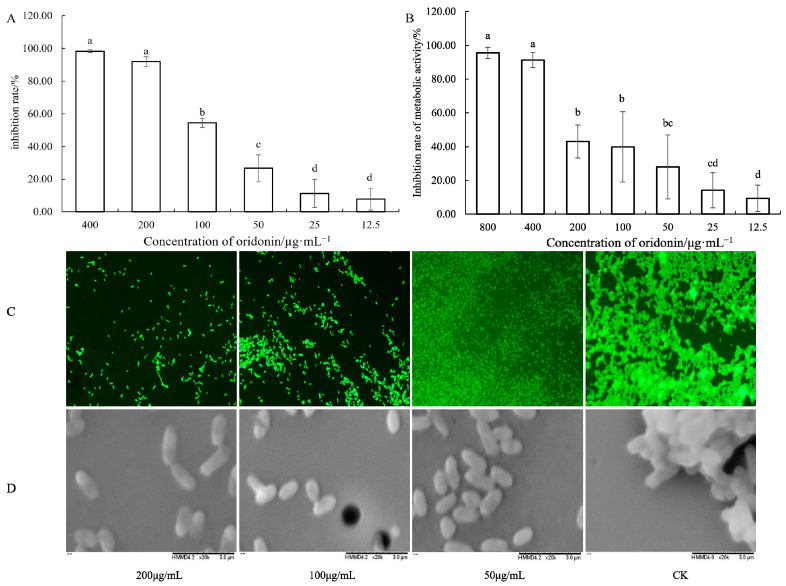
Effects of oridonin on the biofilm formation of *A. hydrophila* AS 1.1801. Notes: (**A**) percentage inhibition of biofilm biomass; (**B**) percentage inhibition of metabolic activity; (**C**) fluorescence microscopy images (1600×); (**D**) scanning electron microscopy images (20,000×). Data are presented as the mean ± SD of three independent experiments. The results were analyzed with one-way ANOVA using Duncan’s multiple range test. a–d: Values with different letters are significantly different (*p* < 0.05), while those with similar letters are not.

In a crystal violet (CV) assay, the biofilm formation of *A. hydrophila* AS 1.1801 was inhibited in a dose-dependent manner by oridonin treatment (Figure 8A). The maximum biofilm inhibition rate of 98.26% was observed at an oridonin concentration of 4 × MIC. Similar results were observed for the viable cell assays with an inhibition of metabolic activity of 91.81% (Figure 8B).

The inhibitory effects of oridonin on biofilm development were additionally assessed via fluorescence microscopy (Figure 8C) and scanning electron microscopy (Figure 8D). Compared with the blank group, which had a thicker biofilm and more extracellular matrix, the addition of oridonin reduced the number of *A. hydrophila* AS 1.1801 that adhered to coverslips (Figure 8C), consistent with the CV assay results. Visible reductions in biofilm aggregation were also evident in oridonin-treated samples (Figure 8D).

### 3.8. Effect of Oridonin on the Modulation of A. hydrophila AS 1.1801 Virulence Gene Expression

**Figure 9:**
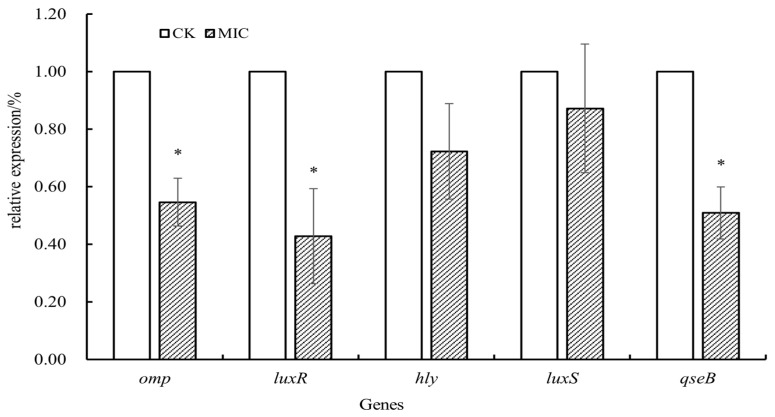
Effect of oridonin on the expression of virulence-related genes in *A. hydrophila* AS 1.1801. Notes: Data are displayed as the mean ± SD. * indicate *p* < 0.05.

The impact of oridonin on the expression of virulence-related genes in *A. hydrophila* AS 1.1801 is depicted in Figure 9. The expression levels of virulence-related genes in *A. hydrophila* AS 1.1801 decreased after 1 × MIC oridonin treatment for 24 h at 28 °C relative to control samples (without oridonin). The expression levels of the *omp* gene and quorum sensing system-related genes (*luxR* and *qseB*) were significantly downregulated (*p* < 0.05). However, there was no significant difference in the expressions of *luxS* and *hly* genes.

## 4. Discussion

*Aeromonas* species are one of the most important etiologies of diseases in fish farms, leading to clinical manifestation and mortality and are associated with public health risks [9]. However, the use of antibiotics against this disease has attracted increasing awareness of drug resistance and food safety. Natural plant-derived antimicrobial substances possess good antibacterial activity, suggesting that they may be an alternative to antibiotics against *A. hydrophila* [12,13,14,15,16,17].

Previous studies have shown that oridonin possessed significant antibacterial activity [23,34,35].The present study determined that the MIC of oridonin against *A. hydrophila* AS 1.1801 was 100 μg/mL. Other investigations reported the MIC of gallic acid [36] and cinnamaldehyde [12] against *A. hydrophila* were 125 μg/mL and 128 μg/mL, respectively. In comparison with gallic acid and cinnamaldehyde, oridonin exhibits superior antibacterial efficacy.

The cell membrane, primarily composed of phospholipids and proteins, plays a vital role in maintaining the integrity and permeability crucial for material, energy and information exchange between cells and the environment [37]. Antibacterial substances can kill or inhibit microorganisms by destroying membrane structure, altering membrane potential, increasing membrane permeability, and causing intracellular material leakage [38]. The importance of cell membrane integrity for normal cell function and, indeed, survival is well established. In the present study, the relative electric conductivity assay results showed that oridonin at inhibitory concentration and subinhibitory concentration could apparently enhance the conductivity of the cultures.

The maintenance of membranous permeability, safeguarding bacteria from harmful agents while facilitating the influx of nutrients essential for bacterial growth, is crucial for normal cell function. The bacterial cell wall and its membranes can restrict the movement of large molecules of DNA in the cell. Experimental results showed that the amount of DNA and RNA released from the cells increased remarkably after *A. hydrophila* AS 1.1801 was treated with different concentrations of oridonin for 3 h. So, it could be conferred that the cytoplasmic leakage caused by the turbulence of membranous permeability would be an important factor in inhibiting bacterial growth. This observation is in accord with the results for changes in cell membrane permeability of oridonin against methicillin-resistant *S. aureus* [23].

Additionally, the experimental findings indicated that the synthesis of intracellular proteins and nucleic acids in *A. hydrophila* AS 1.1801 might be suppressed by oridonin. The results of intracellular soluble protein content assays showed a significant decrease in the protein content of the bacteria treated with inhibitory concentration oridonin for 3 h. Fluorescence microscope observations revealed that the oridonin treatment group at various concentrations exhibited weak fluorescence intensity and low fluorescence density due to low DNA and RNA contents in the cytoplasm. These results align well with the growth and kill-time analysis of oridonin against *A. hydrophila* AS 1.1801. Notably, subinhibitory concentrations of oridonin exerted a pronounced concentration-dependent effect on the duration of the lag phase for *A. hydrophila* AS 1.1801 tested. The delay in the onset of growth caused by exposure to oridonin can only be explained by the inhibition of protein and nucleic acid.

Ultrastructural studies revealed that the impacts of oridonin on *A. hydrophila* AS 1.1801 were mainly cytoplasm agglutination and cytoplasmic structure, as revealed by TEM. Some cells lost their shapes and general morphological integrity after oridonin treatment. The distribution of cytoplasmic components became nonuniform, with the cytoplasm exhibiting aggregations and vacuoles corresponding to black and bright regions, respectively. The numerous rough structures on the cell surfaces were identified as vesicles, potentially cellular constituents leaking from the cells during oridonin treatment, indicative of increased permeability of cell membranes. A study by Zhang et al. (2011) [39] similarly reported changes in the surface structure, membrane penetration, and cytoplasmic structure of *Pseudomonas aeruginosa* cells observed through transmission electron micrographs. The results of TEM simultaneously revealed the effect on the fission of *A. hydrophila* AS 1.1801 cells. This observation is similar to the ultrastructural changes in MRSA cells observed by Yuan et al. (2019) [23].

The pathogen produces various virulence factors, including hemolysins, lipase, protease and others. Hemolysins have been extensively studied in various bacterial species. Pathogenic *A. hydrophila* typically produces two hemolytic toxins: hemolysin and aerolysin. The virulence strains of *A. hydrophila* isolated from the clinic and environment carry the *hlyA* or *aerA* gene [40]. The strains positive for both genes may be highly virulent [41]. This study observed that the hemolytic activity of *A. hydrophila* AS 1.1801 was inhibited at an oridonin concentration higher than 50 μg/mL. However, the results from qRT-PCR indicated that the expression of the *hly* gene in *A. hydrophila* AS 1.1801 treated with 1 × MIC oridonin was not significantly different compared with the control group.

In addition to hemolysin, *A. hydrophila* may also produce other toxins as important factors in pathogenesis, such as extracellular protease and lipase. The extracellular proteases secreted by pathogenic bacteria are important regulators of invasiveness and the establishment of infection as they can overcome initial host defense mechanisms and provide the nutrients needed to allow cells to proliferate [41,42]. The present study demonstrates that oridonin at 1/2 × MIC could significantly decrease the lipase activities, and oridonin at 1/4 × MIC could significantly decrease the extracellular protease activities of *A. hydrophila* AS 1.1801 (*p* < 0.05). The experiment results also confirmed that the treatment could be more effective with a higher dose. This is consistent with prior reports by Li et al. (2018) [43] using cinnamaldehyde, an active component in cinnamon, which was found to inhibit protease production by *Pseudomonas fluorescens* in a dose-dependent manner.

Motility is widely acknowledged as an important virulence factor for pathogenic bacteria, enabling these microbes to move into better environments when nutrients become scarce and to compete with other microbes. The effects of oridonin on *A. hydrophila* AS 1.1801 motility were determined first because, in pathogenic bacteria, flagella-mediated swarming motility is important for substrate adhesion, biofilm formation, and host invasion [44,45]. The diameters of bacterial halos in the presence of 1/2 × MIC, 1 × MIC, and 2 × MIC oridonin were significantly reduced relative to the control treatment (*p* < 0.05), thereby demonstrating that oridonin can reduce the swarming motility of *A. hydrophila* AS 1.1801.

Biofilm is a multicellular structure containing microorganisms growing on the surface of some carriers by adhesion and wrapped with their own secreted extracellular polymeric substances (EPSs) [46]. Biofilms can improve the ability of bacteria to grow and survive by better protecting them against antimicrobial compounds and host immunity while improving nutrient availability, with such biofilm formation being closely linked to pathogenicity [47]. Biofilms are thought to induce roughly 65% of all infections among hospitalized individuals, with the tenacity of these biofilms making the eradication of these infections very difficult [48]. Herein, it was found that oridonin at a concentration of 1 × MIC was effective in inhibiting the biofilm formation of *A. hydrophila* AS 1.1801, and the inhibition rate was 54.44%. With the increase in oridonin content from 1 × MIC to 2 × MIC, the inhibition rate reached 91.81%. Fluorescence microscopy and SEM images also showed that oridonin significantly inhibited the biofilm formation of *A. hydrophila* AS 1.1801. Fluorescence microscopy imaging of *A. hydrophila* AS 1.1801 biofilms treated with 2 × MIC oridonin exhibited only a few isolated bacterial colonies instead of a recognizable biofilm structure. The SEM images of *A. hydrophila* AS 1.1801 biofilm treated with 2 × MIC oridonin also showed that only a few scattered bacterial cells were noted. However, the blank control biofilms were densely colonized hierarchically and three-dimensionally. Because biomass quantitation does not distinguish between live and dead cells, MTT assays were used to evaluate the effect of oridonin on inactivate *A. hydrophila* AS 1.1801 cells in biofilms, and it was found that the inhibition rate of metabolically active *A. hydrophila* AS 1.1801 decreased after oridonin treatment, which was negatively correlated with oridonin concentrations. After treating the biofilm with a 4 × MIC concentration of oridonin, 91.31% of the bacteria lost their metabolic activity.

In the present study, qRT-PCR analysis of the transcript levels of virulence-related genes of *A. hydrophila* AS 1.1801 treated with 1 × MIC oridonin showed that the *omp* gene was significantly down-regulated compared with the control (*p* < 0.05). Outer membrane proteins (OMPs) are the main structure of the outer membrane of Gram-negative bacteria, which play an important role both in bacterial pathogenesis and as a source of antigens. *Acinetobacter baumannii* OmpA performs a range of important functions, including structural roles, antimicrobial activities, and activities associated with virulence, including host cell adhesion, biofilm formation, cytotoxicity, and fibronectin binding [49].

*A. hydrophila* has complex quorum sensing regulation systems, such as *luxR*/*I*, *luxS*/AI-2, and *qseBC*, which can co-regulate the growth, motility, virulence genes expression and biofilm formation of cell population according to the signal molecules in the surrounding environment, and plays an important role in bacterial pathogenicity [20]. Some research studies reported that the *luxS* and *ahyRI* mutants altered key motility and biofilm formation-related genes in wild-type *A. hydrophila* [50,51]. Kozlova et al. (2012) [52] found that deleting the QseBC two-component signal transduction system disrupted *A. hydrophila* pathogenicity in a murine model of septicemia. The results of this study showed that the *luxR* and *qseB* genes of *A. hydrophila* AS 1.1801 treated with 1 × MIC oridonin were significantly downregulated (*p* < 0.05). This suggests that oridonin may affect the expression of virulence factors in *A. hydrophila* AS 1.1801, such as hemolytic activity and biofilm formation, by inhibiting the gene expression of the quorum sensing regulatory system, which needs to be further confirmed through further experiments.

## 5. Conclusions

In summary, these findings substantiated the antibacterial and anti-virulence potential of oridonin against *A. hydrophila* AS 1.1801. Oridonin could cause irreversible damage to the cell membrane of *A. hydrophila* AS 1.1801, resulting in the leakage of nucleic acids, and inhibited cell proliferation by interfering with protein synthesis and nucleic acid replication simultaneously, eventually leading to cell death. Inhibitory concentrations of oridonin could inhibit the production of virulence factors (biofilm, motility, hemolysin, lipase, and protease) and downregulate the expression of virulence-related genes (*luxR*, *qseB* and *omp*) in *A. hydrophila*. Oridonin is expected to become an alternative for the treatment of infectious diseases caused by bacteria in aquaculture.

## Figures and Tables

**Table 1:** Primers used for quantitative PCR.

Primer	Sequence (5′-3′)
*rpob*-F	GGATCACGGTGCCTACAT
*rpob*-R	TAACGCTCGGAAGAGAAGA
*hly* F	TCCGCACTATCTTGGCATCC
*hly* R	TCTACCTCAACGTCAACCGC
*omp*-F	GCAGAACAAACCGCTCAAGG
*omp*-R	CGATCAGGGATTCGGTGGAG
*luxR*-F	GCAGACCCCGAACAAGGACACC
*luxR*-R	CACGCATGAAACCGGCAAACAG
*luxS*-F	GACTTCGGGGTGAGAAACGG
*luxS*-R	AAGATGTAGCTGGCGCAGAA
*qseB*-F	CCAGTCTTCCCGCATCACCAAC
*qseB*-R	GAGCTCCCTGTCCTCTCCCCGC

**Table 2:** Extracellular DNA and RNA content of *A. hydrophila* AS 1.1801 by treatment with oridonin.

Concentration of Oridoninμg/mL	*OD*_260_			
	0 h	3 h	6 h	9 h
0	0.092 ± 0.006 ^a^	0.104 ± 0.004 ^a^	0.109 ± 0.014 ^a^	0.126 ± 0.006 ^a^
1/4 × MIC	0.112 ± 0.003 ^a^	0.391 ± 0.059 ^b^	0.403 ± 0.055 ^b^	0.419 ± 0.069 ^b^
1/2 × MIC	0.124 ± 0.015 ^a^	0.532 ± 0.043 ^b^	0.553 ± 0.043 ^b^	0.561 ± 0.047 ^b^
1 × MIC	0.141 ± 0.026 ^a^	1.010 ± 0.140 ^c^	1.014 ± 0.137 ^c^	1.032 ± 0.127 ^c^
2 × MIC	0.092 ± 0.074 ^a^	1.822 ± 0.186 ^d^	1.827 ± 0.164 ^d^	1.832 ± 0.165 ^d^

Notes: Data are presented as the mean ± SD of three independent experiments. The results were analyzed with one-way ANOVA using Duncan’s multiple range test. a–d: Values with different letters are significantly different (*p* < 0.01), while those with similar letters are not.

**Table 3:** Content of intracellular soluble protein of *A. hydrophila* AS 1.1801 treated with oridonin.

Concentration of Oridonin μg/mL	Concentration of Intracellular Liquid Protein (μg/mL)		
	3 h	6 h	9 h
0	1075.85 ± 10.60 ^a^	1092.02 ± 11.32 ^a^	1026.64 ± 42.10 ^a^
1/4 × MIC	936.15 ± 24.35 ^b^	836.71 ± 185.21 ^b^	1019.07 ± 123.98 ^a^
1/2 × MIC	895.89 ± 64.169 ^b^	1005.31 ± 513.46 ^a^	880.06 ± 41.94 ^b^
1 × MIC	671.55 ± 90.36 ^c^	672.58 ± 84.66 ^d^	745.18 ± 84.08 ^b^
2 × MIC	669.14 ± 102.87 ^c^	742.43 ± 29.35 ^bd^	807.81 ± 21.08 ^b^

Notes: Data are presented as the mean ± SD of three independent experiments. The results were analyzed with one-way ANOVA using Duncan’s multiple range test. a–d: Values with different letters are significantly different (*p* < 0.05), while those with similar letters are not.

## Data Availability

The data presented in this study are available within the article.
